# Post-contrast acute kidney injury. Part 2: risk stratification, role of hydration and other prophylactic measures, patients taking metformin and chronic dialysis patients: Recommendations for updated ESUR Contrast Medium Safety Committee guidelines

**DOI:** 10.1007/s00330-017-5247-4

**Published:** 2018-02-07

**Authors:** Aart J. van der Molen, Peter Reimer, Ilona A. Dekkers, Georg Bongartz, Marie-France Bellin, Michele Bertolotto, Olivier Clement, Gertraud Heinz-Peer, Fulvio Stacul, Judith A. W. Webb, Henrik S. Thomsen

**Affiliations:** 1 Department of Radiology, C2-S, Leiden University Medical Center, Albinusdreef 2, NL-2333 ZA Leiden, The Netherlands; 2 Institute for Diagnostic and Interventional Radiology Klinikum Karlsruhe, Moltkestraße 90, D-76133 Karlsruhe, Germany; 3 Department of Diagnostic Radiology, University Hospitals of Basel, Petersgaben 4, CH-4033 Basel, Switzerland; 4 Service Central de Radiologie Hôpital Paul Brousse 14, av. P.-V.-Couturier, F-94807 Villejuif, France; 5 Department of Radiology, University of Trieste, Strada di Fiume 447, I-34149 Trieste, Italy; 6 Department of Radiology, Assistance Publique-Hôpitaux de Paris, Hôpital Européen Georges Pompidou, 20 rue Leblanc, Paris Cedex 15, F-71015 Paris, France; 7 Department of Radiology, Zentralinstitut für medizinische Radiologie, Diagnostik und Intervention, Landesklinikum St. Pölten, Propst Führer-Straße 4, AT-3100 St. Pölten, Austria; 8 S.C. Radiologia Ospedale Maggiore, Piazza Ospitale 1, I-34129 Trieste, Italy; 9 Department of Radiology, St. Bartholomew’s Hospital, University of London, West Smithfield, EC1A 7BE, London, UK; 10 Department of Diagnostic Radiology 54E2, Copenhagen University Hospital Herlev, Herlev Ringvej 75, DK-2730 Herlev, Denmark

**Keywords:** Contrast media, Acute kidney injury, Metformin, Haemodialysis, Practice guidelines

## Abstract

**Objectives:**

The Contrast Media Safety Committee (CMSC) of the European Society of Urogenital Radiology (ESUR) has updated its 2011 guidelines on the prevention of post-contrast acute kidney injury (PC-AKI). The results of the literature review and the recommendations based on it, which were used to prepare the new guidelines, are presented in two papers.

**Areas covered in part 2:**

Topics reviewed include stratification of PC-AKI risk, the need to withdraw nephrotoxic medication, PC-AKI prophylaxis with hydration or drugs, the use of metformin in diabetic patients receiving contrast medium and the need to alter dialysis schedules in patients receiving contrast medium.

**Key points:**

*• In CKD, hydration reduces the PC-AKI risk*

*• Intravenous normal saline and intravenous sodium bicarbonate provide equally effective prophylaxis*

*• No drugs have been consistently shown to reduce the risk of PC-AKI*

*• Stop metformin from the time of contrast medium administration if eGFR < 30 ml/min/1.73 m* ^*2*^

*• Dialysis schedules need not change when intravascular contrast medium is given*

## Introduction

The Contrast Media Safety Committee (CMSC) of the European Society of Urogenital Radiology (ESUR) produced their most recent guidelines on what was then termed contrast-induced nephropathy (CIN) in 2011 [1]. Guidelines on the use of contrast media (CM) in patients on dialysis and on the use of CM in diabetic patients using metformin were published in 2002 and 2014 [2, 3]. This review provides recommendations for updating the CMSC guidelines which were obtained using a structured literature review based on clinical questions and Patient–Intervention–Comparator–Outcome (PICO) formatting. Since the literature related to the topics considered is so large, the results of the review have been split into two papers. The review only considers post-contrast kidney injury (PC-AKI) after iodine-based CM because acute kidney injury is not associated with gadolinium-based contrast agents in doses approved for clinical magnetic resonance imaging.

In this second paper on PC-AKI, the following topics related to patient management are considered:

1. The role of questionnaires and risk scores to identify at-risk patients with reduced renal function
2. The need to stop nephrotoxic medication before giving CM
3. The optimal hydration protocols for protecting against PC-AKI
4. The possible role of prophylactic drug treatment in preventing PC-AKI
5. The need to adapt metformin administration when giving CM
6. The need to alter schedules for dialysis in the period before and after CM administration

Recommendations are made for items 1–6. The recommendations have been incorporated into version 10 of the ESUR CMSC guidelines, at the end of this paper (Table 4).

## Materials and methods

The recommendations were prepared using the Appraisal of Guidelines for Research and Evaluation (AGREE) II document [4]. A guideline Writing Group (WG) prepared ten clinical questions in PICO format [5]. Systematic search strings were developed with a professional librarian for four different biomedical literature databases (PubMed, Web of Science, Embase and the Cochrane Library). The titles and abstracts were screened for relevance and selected on predefined inclusion and exclusion criteria. Emphasis was put on comparative studies with strong scientific evidence, such as meta-analyses and systematic reviews, and prospective randomised controlled trials (RCTs). The six systematic searches in this manuscript yielded 3402 references of which 445 were selected on the basis of title and abstract. After review of the full text of these 445 publications, 145 were selected for inclusion in this paper. The quality of the evidence from the selected articles was evaluated according to the Oxford Centre for Evidence Based Medicine levels of evidence: grade A, established scientific evidence; grade B, scientific presumption; grade C, low level of evidence [6]. When there was no scientific evidence, recommendations were based on WG consensus and were graded as expert opinion (grade D).

The full description of the materials and methods appears in part 1.

The term intra-arterial injection with first pass renal exposure indicates that contrast medium reaches the kidneys in a relatively undiluted form, e.g. injection into the left heart, thoracic and suprarenal abdominal aorta or the renal arteries. The term intra-arterial injection with second pass renal exposure indicates that contrast medium reaches the renal arteries after dilution either in the pulmonary or peripheral circulation, e.g. injection into the right heart, pulmonary artery, carotid, subclavian, coronary, mesenteric or infrarenal arteries.

## Results

### Question 5: Should questionnaires or scoring systems be used for risk stratification by clinicians when they request a contrast-enhanced imaging study?

#### Patient questionnaires

Questionnaires, such as that proposed by Choyke [7], use information about a history of renal disease or renal surgery, heart failure, diabetes, proteinuria, hypertension and gout to stratify patients for their PC-AKI risk so that sCr measurements need only be done selectively. This may work well and can save resources [8]. Observational studies have shown that these questionnaires can identify patients with eGFR < 45 ml/min/1.73 m^2^ with adequate sensitivity, especially if they are aged less than 70 [9–11]. Since eGFR measurement can detect more patients with renal dysfunction than questionnaires [12], with easier patient logistics and similar cost-effectiveness [13, 14], many hospitals have adopted a policy of sCr measurements in *all* patients scheduled for intravenous (IV) CM and no longer use questionnaires for risk stratification or selection for eGFR measurement (Table 1).

**Table 1:** PC-AKI: Risk stratification; use of nephrotoxic medication

**Risk stratification**
In hospitals which use sCr measurements for all patients before intravascular CM administration there is no benefit in using questionnaires for PC-AKI risk stratification.
In hospitals which use sCr measurements selectively, Choyke questionnaires may be used to identify patients with eGFR < 45 ml/min/1.73 m^2^ before intra-arterial CM administration with first pass renal exposure.
Level of evidence D
Risk prediction scores are only available for coronary angiography and/or percutaneous coronary intervention, and have only modest abilities, so cannot be recommended to stratify the risk of PC-AKI.
Level of evidence A
**Nephrotoxic medication**
In CKD patients receiving CM, optimal nephrologic care involves minimising the use of nephrotoxic drugs.
Level of evidence D
ACE inhibitors and angiotensin receptor blockers do not have to be stopped before CM administration.
Level of evidence B
There is insufficient evidence to recommend withholding nephrotoxic drugs such as NSAIDs, antimicrobial agents or chemotherapeutic agents before CM administration.
Level of evidence C

#### Risk prediction models

No risk models have been produced yet for IV or IA CM administration with second pass renal exposure. For patients having coronary angiography (CA) or percutaneous coronary intervention (PCI), many different risk scores have been proposed to stratify the patient’s PC-AKI risk, and most include pre-procedural and procedural data. A model with only pre-procedural data [15] would be more practical for selecting suitable preventive measures. Risk scores should be verified in relation to improvements in clinical outcome. In clinical practice, a prediction rule would require a high discriminatory value, i.e. a C-statistic greater than 0.80 [16].

The best-known risk model is the eight-variable Mehran score [17], which has been studied in more than 15,000 patients and has been externally validated in multiple studies, but with variable C-statistic values of 0.57–0.85 [16, 18–20]. The Mehran score correlates relatively well with clinical outcomes [21]. Newer risk scores with good discriminatory value, available in user-friendly calculators or smartphone applications, still need external validation [22]. A recent systematic review of 16 risk models concluded that they had only modest predictive value [23] and a review and meta-analysis of 74 risk models noted their heterogeneity and concluded that further research was needed to evaluate the effect of such models on clinical care [24] (Table 1).

### Question 6: Should nephrotoxic medication be withheld to reduce the risk of PC-AKI?

Optimal nephrologic care involves minimizing the use of nephrotoxic drugs where clinically possible [25]. Many frequently prescribed medications, such as nonselective NSAIDs, selective Cox-2 inhibitors, several classes of antimicrobial agents and chemotherapeutic agents have nephrotoxic potential and can induce AKI [26].

There is little good quality data about the relationship between these drugs and PC-AKI [27]. A retrospective cohort study showed that concurrent use of four or more nephrotoxic agents was significantly predictive for PC-AKI in patients given IV CM [28]. A meta-analysis of PC-AKI incidence following CM-enhanced CT found that concurrent administration of NSAIDs was an independent risk factor for PC-AKI [29].

The effect of withholding angiotensin converting enzyme inhibitors (ACEI) and angiotensin-2 receptor blockers (ARB) in chronic users has been extensively evaluated. Multiple RCTs [30, 31] and observational studies gave conflicting results and are limited by small sample sizes and significant heterogeneity [32–34]. However, meta-analyses of RCTs found no lower risk [34]. Withholding ACEI/ARB may be associated with a slightly lower risk of PC-AKI but the evidence is not sufficiently strong to recommend this (Table 1).

### Question 7: What are the most cost- and time-effective protocols for oral and intravenous hydration to reduce the risk of PC-AKI?

#### Hydration as a preventive strategy for PC-AKI

Evidence for prevention of PC-AKI with IV saline hydration (volume expansion) comes from RCTs in patients who received intra-arterial (IA) CM during percutaneous intervention [35–37], and in patients who received bicarbonate hydration before IV enhanced emergency CTPA [38]. One RCT evaluated the evidence for IA CM administration during CA [39]. These studies found that, for both IA and IV CM administration, the incidence of PC-AKI was significantly lower in patients who received IV hydration compared to placebo, and that hydration prevented emergency dialysis [36]. Significant differences for mortality or other adverse events were not found. There were few patients with severe renal impairment (eGFR < 30 ml/min/1.73 m^2^) in almost all studies. The recent AMACING trial showed that for patients with eGFR > 30 ml/min/1.73 m^2^ receiving IV CM there was no difference between no hydration and hydration in preventing PC-AKI [40].

#### Oral hydration versus intravenous saline hydration

Oral intake of clear fluids by patients as an alternative to IV saline to prevent PC-AKI is difficult to monitor or control. Nine studies evaluated oral hydration, but were limited by small patient numbers and by the absence of patients with severe renal impairment [39, 41–48]. Three meta-analyses concluded that there is no evidence that oral hydration is associated with more risk of PC-AKI compared to IV hydration, but the studies were limited by heterogeneity and lack of hard clinical outcomes [49–51]. The CMSC does not recommend the use of oral hydration as the sole preventive strategy for PC-AKI, but unrestricted intake of clear oral fluids in addition to IV volume expansion is supported (Table 2).

**Table 2:** PC-AKI prophylaxis: Hydration, drugs, renal replacement therapy

**Hydration**
Preventive hydration should be used to reduce the incidence of PC-AKI in at-risk patients.
Level of evidence B
Intravenous saline and bicarbonate protocols have similar efficacy for hydration.
Level of evidence A
For intravenous and intra-arterial CM administration with second pass renal exposure hydrate the patient with *either* (a) 3 ml/kg/h bicarbonate 1.4% (or 154 mmol/l solution) for 1 h before CM *or* (b) 1 ml/kg/h saline 0.9% for 3–4 h before and 4–6 h after CM.
Level of evidence D
For intra-arterial CM administration with first pass renal exposure hydrate the patient with *either* (a) 3 ml/kg/h bicarbonate 1.4% (or 154 mmol/l solution) for 1 h before CM followed by 1 ml/kg/h bicarbonate 1.4% (or 154 mmol/l) for 4–6 h after CM
*or* (b) 1 ml/kg/h saline 0.9% for 3–4 h before and 4–6 h after CM.
Level of evidence D
Oral hydration as the sole means of prevention is not recommended.
Level of evidence D
In patients with severe heart failure (NYHA grade 3–4) or patients with end-stage renal failure (CKD grade V) preventive IV hydration should be individualized by the clinician responsible for patient care.
Level of evidence D
**Drugs**
*N*-Acetylcysteine has not been conclusively shown to reduce the risk of PC-AKI in patients with eGFR < 45 ml/min/1.73 m^2^ receiving intravenous or intra-arterial CM, and its use is NOT recommended.
Level of evidence A
Giving short-term, high-dose statins to patients not already taking statins has not been shown to reduce the risk of PC-AKI in patients with eGFR < 45 ml/min/1.73 m^2^ receiving intravenous or intra-arterial CM, and its use is NOT recommended.
Level of evidence B
ACE inhibitors or angiotensin receptor blockers have not been shown conclusively to reduce the risk of PC-AKI in patients receiving intravenous or intra-arterial CM, and their use is NOT recommended.
Level of evidence B
Vitamin C has not been shown conclusively to reduce the risk of PC-AKI in patients receiving intravenous or intra-arterial CM, and its use is NOT recommended.
Level of evidence B
**Renal replacement therapy**
Renal replacement therapy has not been shown conclusively to reduce the risk of PC-AKI in patients receiving intravenous or intra-arterial CM, and its use is NOT recommended.
Level of evidence B

#### Intravenous hydration: saline versus bicarbonate

Normal saline (NaCl 0.9%) and sodium bicarbonate solution (1.4% or 154 mmol NaHCO_3_ in D5W) are the two most commonly studied crystalloid solutions. The rationale for using bicarbonate is that alkalinisation can reduce the formation of free reactive oxygen species [52]. Initial studies favoured bicarbonate [53–56], but this was not replicated in later studies [57–62], so IV hydration with bicarbonate can be considered equivalent to normal saline.

There is no consensus on the optimal hydration regime. Most studies have compared bicarbonate given pre- and post-CM for less than 6 h [53] to longer duration saline pre- and post-CM protocols (12–24 h). In all studies, there are few patients with eGFR < 30 ml/min/1.73 m^2^, and evidence about whether short duration bicarbonate is better than long duration saline is conflicting [63–71]. There is limited evidence on whether pre-hydration only is inferior to pre- and post-hydration, and only one very short duration bicarbonate protocol has been evaluated [38, 72, 73].

Most studies have been performed in cardiac patients admitted for CA or PCI. Three studies evaluated hydration protocols in patients having contrast-enhanced CT, and did not favour bicarbonate over saline [38, 72, 74]. No studies were identified assessing the beneficial effect of other crystalloids. However, balanced crystalloid solutions, such as Ringer’s lactate, may be preferable in critical care populations, because they avoid the harmful effects of hyperchloraemic acidosis.

The CMSC considers that for IV and IA CM injection with second pass renal exposure either a short bicarbonate hydration regime before CM or a conventional protocol with saline given before and after CM may be used. For IA CM injection with first pass renal exposure conventional protocols with either bicarbonate or saline given before and after CM should be used (Table 2).

#### Forced diuresis versus conventional hydration

Newer approaches for patients with impaired left ventricular function combine controlled saline hydration with a forced high urinary flow rate to maintain euvolemia and avoid overhydration and several RCTs showed better results than conventional hydration protocols [75–77]. Other catheter-based strategies used left ventricular end-diastolic pressure or central venous pressure to guide hydration [78, 79]. In these RCTs the incidence of PC-AKI was lower than with standard IV hydration. Since the forced diuresis studies have heterogeneous populations, interventions and control hydration protocols, their findings cannot be pooled. The CMSC considers that there is not sufficient evidence to recommend forced diuresis.

#### In which patients should the hydration protocol be individualized?

There is no data to suggest that patients with severe renal impairment (CKD grade V) or severe heart failure (NYHA grade 3–4) should receive different hydration protocols. However, IV hydration with large volumes may exacerbate acute heart failure and induce pulmonary oedema [40]. The opinion of the CMSC is that hydration protocols in these patients should be individualized for type, volume and duration.

### Question 8: Which other strategies (pharmaceutical, vitamin, renal replacement therapy) have been proved effective in preventing PC-AKI?

#### N-Acetylcysteine (NAc)

Most recent RCTs or meta-analyses do not show a protective effect of NAc against PC-AKI following coronary or peripheral angiography [66, 80–84]. NAc also failed to affect clinical outcome in coronary or peripheral angiography [85] or to have a protective effect in CT [86, 87] or in patients with diabetes mellitus undergoing coronary or peripheral angiography [88, 89]. Comparative studies with NAc combined with saline or sodium bicarbonate protocols did not show any additional effect of protective effect of NAc [61, 90–93]. However, more recent meta-analyses showed a benefit of NAc, with or without high-dose statins, when added to hydration for preventing PC-AKI [94–96].

#### Statins

Several meta-analyses showed lower overall PC-AKI rates with the use of high-dose, short-term statin treatment compared to controls [95–105]. Lower PC-AKI rates were also found in subgroups, such as older patients, patients with acute coronary syndromes and for high-dose statin regimes. Some of these meta-analyses showed a reduced need for RRT after statins, but no reduction in all-cause mortality [97, 102]. However, the US Agency for Healthcare Research and Quality (AHRQ) meta-analysis showed that the risk of PC-AKI was only significantly reduced when statins were added to hydration and NAc. A reduction in PC-AKI risk could not be shown when statins plus hydration were compared to hydration alone in patients not taking statins. The standard of evidence grade was low in both analyses [94].

Despite the many positive results, it is difficult to make a general recommendation for statins [106] because the patients studied were invariably cardiac, and a variety of statin and hydration protocols were used. Patients with CKD grade 3B–5 (eGFR < 45 ml/min/1.73 m^2^) are under-represented in the studies and results in these patients remain inconclusive [102, 103, 107, 108]. Most patients undergoing CA/PCI are already taking long-term statins, and results in these patients are unclear.

While the CMSC recognises the potential preventive effects of short-term statins, it does not advise the use of short-term, high-dose statins as a single strategy for preventing PC-AKI (Table 2).

#### RAAS blockade: ACE inhibitors and angiotensin-II receptor blockers

Administration of renin–angiotensin–aldosterone system (RAAS) blockade as a preventive measure for patients not taking these drugs did not show a significant effect on the incidence of PC-AKI in recent meta-analyses [32, 34] (Table 2).

#### Vitamin C

The majority of RCTs or meta-analyses do not demonstrate a protective effect of vitamin C against PC-AKI in patients with CKD predominantly undergoing coronary angiography [109–111] or any benefit of the use of vitamin C, NAc or a combination of both over the standard hydration regimen in preventing PC-AKI [112, 113] (Table 2). Combining vitamin C with pentoxifylline also failed to show an advantage [114]. Only two publications [115, 116] have shown a protective effect of vitamin C in patients with CKD undergoing CA.

#### Renal replacement therapy (RRT)

There is no convincing evidence in favour of preventive haemodialysis or RRT alone [117–119] or combined with hydration [120] in patients with CKD, predominantly undergoing CA (Table 2). There is no evidence of an increased risk of permanent anuria in patients on peritoneal dialysis undergoing CA [121]. There is a single study showing better late-stage (day 5–30) renal protection against PC-AKI with simultaneous haemodialysis [122].

#### Miscellaneous

The data on the protective effects of several agents, such as trimatizidine [123, 124], theophylline [95, 125–127], alprostadil [128, 129], nebivolol [130], fenoldopam [131] and iloprost [132], is not conclusive and does not support recommending their use to reduce the risk of PC-AKI.

### Question 9: Should administration of metformin be adapted to reduce the risk of metformin-associated lactic acidosis in patients with type 2 diabetes mellitus scheduled to receive intravascular contrast media?

Metformin is the standard drug for monotherapy of type 2 diabetes mellitus [133]. The effect of CM on the risk of metformin-associated lactic acidosis is indirect, since an episode of AKI following intravascular CM administration may lead to metformin accumulation. The use of metformin in patients with eGFR 30–59 ml/min/1.73 m^2^ is considered safe if doses are reduced appropriately [134, 135]. Limiting the metformin dose to a maximum of 2000 mg/day for eGFR 45–60 ml/min/1.73 m^2^ and to a maximum of 1000 mg/day for eGFR 30–44 ml/min/1.73 m^2^ has been recommended. In patients with eGFR 30–59 ml/min/1.73 m^2^ metformin drug levels remain within therapeutic ranges. For patients with eGFR < 30 ml/min/1.73 m^2^ metformin administration is not approved.

Multiple studies and meta-analyses have shown that the risk of lactic acidosis is very low and linked more to the underlying disease and possible co-morbidities rather than the use of metformin [134, 136, 137]. Because of the lack of published evidence on metformin and CM, early guidelines about the need to stop metformin before intravascular CM were based on consensus, and were strict [138, 139]. As the low risk of lactic acidosis became apparent, guidelines have become less restrictive [3].

Since no new published evidence is available, the CMSC has updated its recommendations based on recent recommendations from the FDA [140], and on guidelines from the ACR and RSTN [14, 141] (Table 3).

**Table 3:** Metformin administration, dialysis schedules

**Metformin administration in patients at risk of PC-AKI**
*Note that these recommendations may deviate from current EMA/FDA recommendations.*
Patients with eGFR > 30 ml/min/1.73 m^2^ and no evidence of AKI receiving either intravenous CM or intra-arterial CM with second pass renal exposure: continue taking metformin normally.
Patients (a) with eGFR < 30 ml/min/1.73 m^2^ receiving either intravenous CM or intra-arterial CM with second pass renal exposure *or* (b) receiving intra-arterial CM with first pass renal exposure *or* (c) with AKI: stop taking metformin from the time of CM administration: measure eGFR within 48 hours and restart metformin if renal function has not changed significantly.
Level of evidence D
**Dialysis schedules in relation to CM administration**
It is not necessary to adapt the timing of intravascular CM administration in relation to the dialysis schedule in patients undergoing chronic dialysis or haemofiltration, but it may be done to minimise volume overload.
Level of evidence D

### Question 10: Should the timing of CM administration be adapted to the schedule of haemodialysis or haemofiltration sessions in patients on renal replacement therapy?

Iodine-based CM can be safely removed by haemodialysis (HD) or haemofiltration (HF). Many factors influence the effectiveness of HD, such as flow rate of blood and dialysate, dialysis membrane permeability, HD duration, and CM characteristics such as molecular size, protein binding, hydrophilicity and electrical charge [142].

Although HF concomitant with radiological procedures has been shown to be feasible and well tolerated [143, 144], the fractional removal of iodine-based CM contrast agents is modest and several HF or HD sessions are needed to remove 95% of the administered CM [143]. Also, there is no evidence for the necessity of emergency HD after administration of iodine-based CM in patients on chronic HD [145]. However, to avoid volume overload, CM administration may be synchronised with scheduled HF or HD (Table 3).

## Conclusion

Assessment of the risk of PC-AKI before intravascular CM is administered is best done by measuring eGFR but the alternative of a questionnaire for patients detects most patients with eGFR less than 45 ml/min/1.73 m^2^. Volume expansion with normal saline or sodium bicarbonate remains the mainstay of PC-AKI prevention, but there is still uncertainty about the optimal protocol. The additional benefit of a number of drugs, such as *N*-acetylcysteine, statins, ACE inhibitors and angiotensin-II receptor blockers, and vitamin C in preventing PC-AKI has not been proved conclusively. Stopping nephrotoxic medications appears to be of limited value in preventing PC-AKI. Recommendations for discontinuing metformin when CM is given have been relaxed and now only apply to patients with eGFR < 30 ml/min/1.73 m^2^ receiving IV CM or IA CM with second pass renal exposure, and to all patients receiving IA CM with first pass renal exposure or who have AKI. There is no need to adapt dialysis schedules in patients being given intravascular CM.

The recommendations made in this paper have been incorporated into the ESUR CMSC guidelines version 10 (Table 4).

**Table 4:** ESUR CMSC guideline (version 10) for post-contrast acute kidney injury (PC-AKI)

**Definitions**	
**Post-contrast acute kidney injury (PC-AKI)** is defined as an increase in serum creatinine ≥ 0.3 mg/dl (or ≥ 26.5 μmol/l), or ≥ 1.5 times baseline, within 48–72 h of intravascular administration of a contrast medium.	
**Intra-arterial injection with first pass renal exposure** indicates that contrast medium reaches the renal arteries in a relatively undiluted form, e.g. injection into the left heart, thoracic and suprarenal abdominal aorta or the renal arteries.	
**Intra-arterial injection with second pass renal exposure** indicates that contrast medium reaches the renal arteries after dilution either in the pulmonary or peripheral circulation e.g. injection into the right heart, pulmonary artery, carotid, subclavian, coronary, mesenteric or infra-renal arteries.	
**Measurement of renal function**	
**• Estimated glomerular filtration rate (eGFR), calculated from the serum creatinine,** is recommended to estimate renal function before administration of contrast medium.	
**• In adults ≥ 18 years, the CKD-EPI formula to estimate GFR is recommended.**	
eGFR (ml/min/1.73 m^2^) =	
Female sCr ≤ 62 μmol/l: 144 × (sCr/62)^−0.329^ × 0.993^Age^	
Female sCr > 62 μmol/l: 144 × (sCr/62)^−1.209^ × 0.993^Age^	
Male sCr ≤ 80 μmol/l: 141 × (sCr/80)^−0.411^ × 0.993^Age^	
Male sCr > 80 μmol/l: 141 × (sCr/80)^−1.209^ × 0.993^Age^	
(sCr in μmol/l; age in years)	
*All equations × 1.159 if African American race*	
**• In children, the revised Schwartz formula to estimate GFR is recommended,**	
eGFR (ml/min/1.73 m^2^) = 36.5 × Length/sCr (sCr in μmol/l; length in cm)	
Note: Neither serum nor plasma creatinine is an ideal indicator of renal function and may miss decreased renal function.	
**Renal adverse reactions to iodine-based contrast media**	
**RISK FACTORS FOR PC-AKI**	
Patient-related	• eGFR less than 45 ml/min/1.73 m^2^ before intra-arterial contrast medium administration with first pass renal exposure or in ICU patients
	• eGFR less than 30 ml/min/1.73 m^2^ before intravenous contrast medium or intra-arterial contrast medium administration with second pass renal exposure
	• Known or suspected acute renal failure
Procedure-related	• Intra-arterial contrast medium administration with first pass renal exposure
	• Large doses of contrast medium given intra-arterially with first pass renal exposure
	• High osmolality contrast media
	• Multiple contrast medium injections within 48-72h
**Time of referral**	
**ELECTIVE EXAMINATION**	
**MEASUREMENT OF RENAL FUNCTION**	
• **Measure eGFR before administering intravascular iodine-based contrast medium**	
either (a) In all patients	
or (b) In patients who have a history of	
- Renal disease (eGFR < 60 ml/min/1.73 m^2^)	
- Kidney surgery	
- Proteinuria	
- Hypertension	
- Hyperuricemia	
- Diabetes mellitus	
• **Timing of eGFR measurement**	
- Within 7 days before contrast medium administration in patients with an acute disease, an acute deterioration of a chronic disease or who are hospital inpatients	
- Within 3 months before contrast medium administration in all other patients	
**EMERGENCY EXAMINATION**	
Identify at-risk patients (see above), if possible:	
• Determine eGFR if the procedure can be deferred until the result is available without harm to the patient.	
• If eGFR cannot be obtained, follow the protocols for patients with eGFR less than 45 ml/min/1.73 m^2^ for intra-arterial administration with first pass renal exposure and eGFR less than 30 ml/min/1.73 m^2^ for intravenous and intra-arterial administration with second pass renal exposure as closely as clinical circumstances permit.	
**Before the examination**	
**ELECTIVE EXAMINATION**	
At-risk patients (see above)	• Consider an alternative imaging method not using iodine-based contrast media
	• Intravenous saline and bicarbonate have similar efficacy for preventive hydration
	• For intravenous contrast media administration and intra-arterial contrast media administration with second pass renal exposure hydrate the patient *either* with intravenous sodium bicarbonate 1.4% (or 154 mmol/l in dextrose 5% water): 3 ml/kg/h for 1 h before contrast medium *or* with intravenous saline 0.9%, 1 ml/kg/h for 3–4 h before and 4–6 h after contrast medium
	• For intra-arterial contrast media administration with first renal exposure hydrate the patient *either* with intravenous sodium bicarbonate 1.4% (or 154 mmol/l in dextrose 5% water): 3 ml/kg/h for 1 h before and 1 ml/kg/h for 4–6 h after contrast medium *or* with intravenous saline 0.9%, 1 ml/kg/h for 3–4 h before and 4–6 h after contrast medium
	• The clinician responsible for patient care should individualize preventive hydration in patients with severe congestive heart failure (NYHA grade 3–4) or patients with end-stage renal failure (eGFR < 15 ml/min/1.73 m^2^)
	• Oral hydration is not recommended as the sole method of preventive hydration
**EMERGENCY EXAMINATION**	
At-risk patients (see above)	• Consider an alternative imaging method not using iodine-based contrast media
	• Use preventive hydration before contrast medium administration (see ‘Elective Examination’ for protocols)
**Time of examination**	
All patients	• Use low or iso-osmolar contrast media
	• Use the lowest dose of contrast medium consistent with a diagnostic result
	• For intra-arterial contrast medium administration with first pass renal exposure keep *either* the ratio CM dose (in gram I)/*absolute* eGFR (in ml/min) < 1.1 *or* the ratio CM volume (in ml)/eGFR (in ml/min/1.73 m^2^) < 3.0 *(assuming a contrast medium concentration of 350 mg iodine/ml)*
**After the examination**	
At-risk patients	• Continue preventive hydration if appropriate (see protocols above)
	• Determine eGFR 48 h after administration of contrast medium
	• If at 48 h there is a diagnosis of PC-AKI, monitor the patient clinically for at least 30 days and determine eGFR at regular intervals
Note: **No pharmacological prophylaxis** (with statins, renal vasodilators, receptor antagonists of endogenous vasoactive mediators or cytoprotective drugs) has been shown to offer consistent protection against PC-AKI.	
**Patients with diabetes mellitus taking metformin**	
• Patients with eGFR > 30 ml/min/1.73 m^2^ and no evidence of AKI receiving either intravenous or intra-arterial iodine-based contrast medium with second pass renal exposure: Continue taking metformin normally.	
• Patients (a) with eGFR < 30 ml/min/1.73 m^2^ receiving either intravenous or intra-arterial contrast medium with second pass renal exposure *or* (b) receiving intra-arterial contrast medium with first pass renal exposure *or* (c) with AKI:	
Stop taking metformin from the time of contrast medium administration. Measure eGFR within 48 h and restart metformin if renal function has not changed significantly.	
**Dialysis and contrast medium administration**	
• All iodine-based contrast media can be removed by haemodialysis or peritoneal dialysis.	
• There is no evidence that haemodialysis protects patients with normal or impaired renal function from PC-AKI.	
• In all patients, avoid osmotic and fluid overload.	
**PATIENTS ON DIALYSIS**	
**Patients on haemodialysis**	• Co-ordinating the time of the iodine-based contrast medium injection with the haemodialysis session is unnecessary
	• Extra haemodialysis session to remove iodine-based contrast medium is unnecessary
**Patients on continuous ambulatory peritoneal dialysis**	Haemodialysis to remove iodine-based contrast medium is unnecessary

## Abbreviations and acronyms

ACEI: Angiotensin Converting Enzyme Inhibitor
ACR: American College of Radiology
AGREE: Appraisal of Guidelines for Research and Evaluation
AHRQ: Agency for Healthcare Research and Quality
AKI: Acute Kidney Injury
ARB: Angiotensin-II Receptor Blocker
CA: Coronary Angiography
CI-AKI: Contrast-Induced Acute Kidney Injury
CIN: Contrast-Induced Nephropathy
CKD: Chronic Kidney Disease
CM: Contrast Media
CMSC: Contrast Media Safety Committee
CT: Computed Tomography
CTPA: Computed Tomography Pulmonary Angiography
D5W: Dextrose 5% in Water
eGFR: Estimated Glomerular Filtration Rate
ESUR: European Society of Urogenital Radiology
FDA: Federal Drugs Administration
GFR: Glomerular Filtration Rate
HD: Haemodialysis
HF: Haemofiltration
IA: Intra-Arterial
IV: Intravenous
NAc: *N*-Acetylcysteine
NaHCO_3_: Sodium Bicarbonate
NSAID: Non-Steroidal Anti-Inflammatory Drug
NYHA: New York Heart Association
OCEBM: Oxford Centre for Evidence Based Medicine
PC-AKI: Post-Contrast Acute Kidney Injury
PCI: Percutaneous Coronary Intervention
PICO: Patient–Intervention–Comparator–Outcome
PS: Propensity Score
RAAS: Renin–Angiotensin–Aldosterone System
RSTN: Radiological Society of the Netherlands
RCT: Randomised Controlled Trial
RRT: Renal Replacement Therapy
sCr: Serum Creatinine
WG: Writing Group
